# SBRT Treatment of Metachronous Small-Cell and Non-Small-Cell Lung Carcinomas in a Patient with Severe COPD

**DOI:** 10.7759/cureus.400

**Published:** 2015-12-11

**Authors:** Adele Duimering, Zsolt Gabos, Brock Debenham

**Affiliations:** 1 Radiation Oncology, Cross Cancer Center, University of Alberta

**Keywords:** small cell lung cancer, non-small cell lung carcinoma, copd, radiotherapy, stereotactic body radiotherapy

## Abstract

Stereotactic body radiotherapy (SBRT) has not been widely employed in the treatment of limited-stage (LS) small-cell lung cancer (SCLC), although SBRT finds particular utility in patients medically unfit to undergo surgical resection or radiotherapy with conventional fields. The authors present the case of a 61-year-old female smoker with severe chronic obstructive pulmonary disease (COPD), diagnosed incidentally with LS-SCLC. Concurrent chemoradiotherapy was contraindicated by her poor pulmonary function, and she was treated radically with four cycles of cisplatin and etoposide chemotherapy. This was followed by prophylactic cranial irradiation and consolidative SBRT (48 Gy in 4 fractions) to the residual tumour, which achieved a complete clinical response. Fifteen months following the patient’s initial diagnosis, a metachronous Stage IA contralateral non-small cell lung cancer (NSCLC) was incidentally diagnosed and was treated with SBRT (48 Gy in 4 fractions). Although studies have established that the incidence of a second lung cancer is higher in patients with previous SCLC, this case is unique in that both primaries were treated with SBRT.

## Introduction

The standard treatment of LS-SCLC in Canada consists of radical combined chemoradiotherapy: four cycles of cisplatin and etoposide, concurrent with thoracic irradiation (40 Gy in 15 fractions to 50 Gy in 25 fractions; or the Turrisi Regimen of 45 Gy in 30 fractions, dosed twice daily). Although the benefits of this concurrent thoracic radiotherapy in improving local control and overall survival were recognized a quarter-century ago, no large studies have compared conventionally fractionated external beam radiotherapy to SBRT in SCLC patients [1].

Fit LS-SCLC patients who lack evidence of nodal involvement or distant metastases may be considered for primary surgical resection, followed by chemotherapy [2]. Patients who have achieved at least disease stability after initial chemoradiotherapy or surgery and chemotherapy are offered prophylactic cranial irradiation (25 Gy in 10 fractions).

Patients surviving SCLC have a 2–13% per year risk of developing a second primary lung cancer, a 7- to 16-fold higher relative risk than a similar North American population [3]. The predominant histology of the second primary in this population is squamous cell carcinoma [3].

Stage I NSCLC is primarily managed by surgical resection, which achieves a locoregional control rate of 90% and five-year overall survival rates of 50–70% [4]. In medically inoperable patients treated with primary radiotherapy, these locoregional control and five-year overall survival rates drop dramatically to 30–70% and 15–30%, respectively. While the overall survival difference may be attributed, in part, to poorer performance statuses of those unfit for surgery, the difference in local control raises the question of whether sufficiently high radiation doses are being prescribed [5]. Dose-limiting toxicities may be avoided by replacing conventionally fractionated radiotherapy with SBRT, thereby permitting higher per fraction radiation doses to be delivered. SBRT has been demonstrated to achieve similar rates of local control to surgical resection and, as such, is a reasonable first-line treatment for medically inoperable Stage I NSCLC that “may even challenge surgery in operable instances” [6-7].

## Case presentation

A 61-year-old female 50-pack-year smoker with severe COPD, advanced emphysema, chronic hypoxia on home oxygen secondary polycythemia, non-insulin-dependent diabetes mellitus, hypertension, dyslipidemia, and a stable, untreated renal cell carcinoma presented with an incidental finding of a left-upper-lobe (LUL) nodule on a chest x-ray performed during a COPD exacerbation. Apart from the chronic dyspnea from her underlying lung disease, the patient was asymptomatic with good functional capacity (Eastern Cooperative Oncology Group score 1). Physical exam was unremarkable. Signed informed patient consent was obtained.

**Figure 1:**
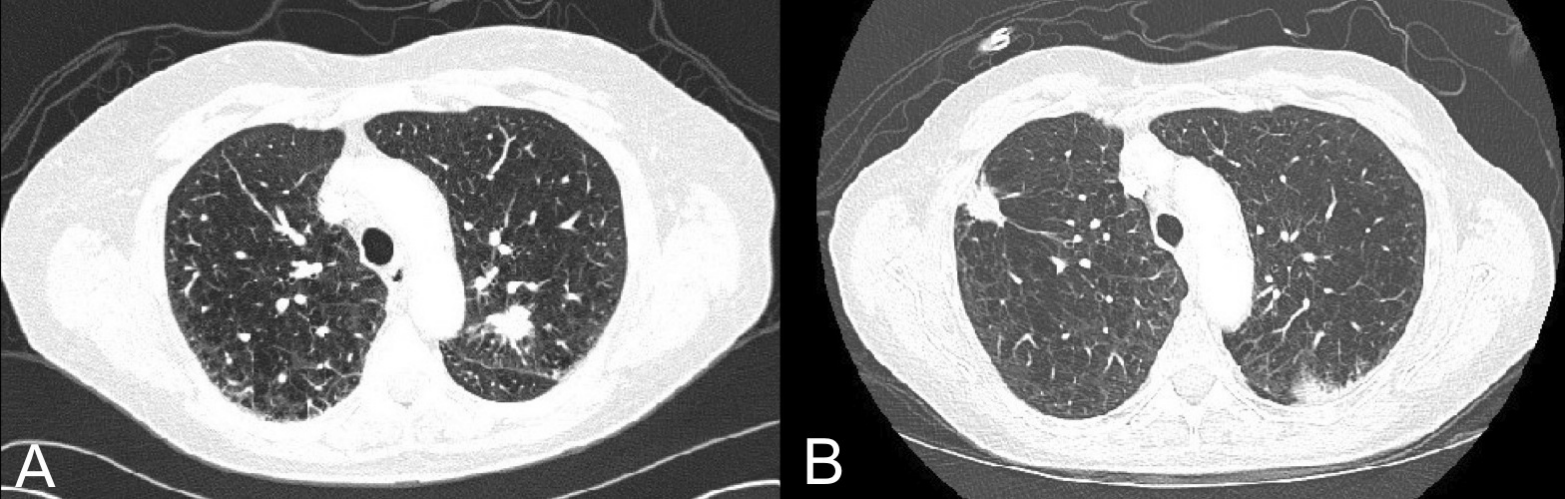
Pre-treatment CT images of (A) LUL LS-SCLC and (B) RUL Stage IA NSCLC.

Further imaging characterized an apical-posterior spiculated noncalcified 1.7 cm FDG-avid LUL pulmonary nodule, with no evidence of nodal involvement or metastatic disease (Figure 1A). Despite initial benign bronchoscopic LUL washing and brushing cytology, fine needle aspiration (FNA) biopsy of the mass demonstrated small-cell carcinoma (cT1a, cN0, cM0, Stage IA).

Given her poor pulmonary function (FEV1 of 0.39 pre-bronchodilator and 0.5 post-bronchodilator, DLCO of 37% predicted), the patient was deemed not to be a candidate for combined chemoradiotherapy. Chemotherapy proceeded with the standard regimen of four cycles of cisplatin and etoposide. CT performed two weeks following chemotherapy completion demonstrated only partial response of the primary lesion, and the patient was offered adjuvant radical irradiation for the residual LUL tumour. An SBRT technique was selected to minimize pulmonary toxicity. Starting eight weeks following the last cycle of chemotherapy, 48 Gy, prescribed to 95% of the PTV, were delivered in four fractions over two weeks by dynamic conformal arcs. Prophylactic cranial irradiation (25 Gy in 10 fractions) was also administered. The patient tolerated this treatment well and interval post-treatment imaging has demonstrated a sustained complete response.

Fifteen months after her initial diagnosis, the patient presented with a COPD exacerbation and hyponatremia. A CT scan revealed a new 2 cm peripheral right-upper-lobe (RUL) pulmonary nodule with FNA cytology demonstrating atypical epithelial cells, suspicious for NSCLC (not further histologically characterizable, given the limited FNA sample; cT1a, cN0, cM0, Stage IA) (Figure 1B). A right middle lobe collapse was noted and was ascribed to mucus bronchial plugging secondary to the patient’s underlying lung disease, rather than malignant obstruction.

**Figure 2:**
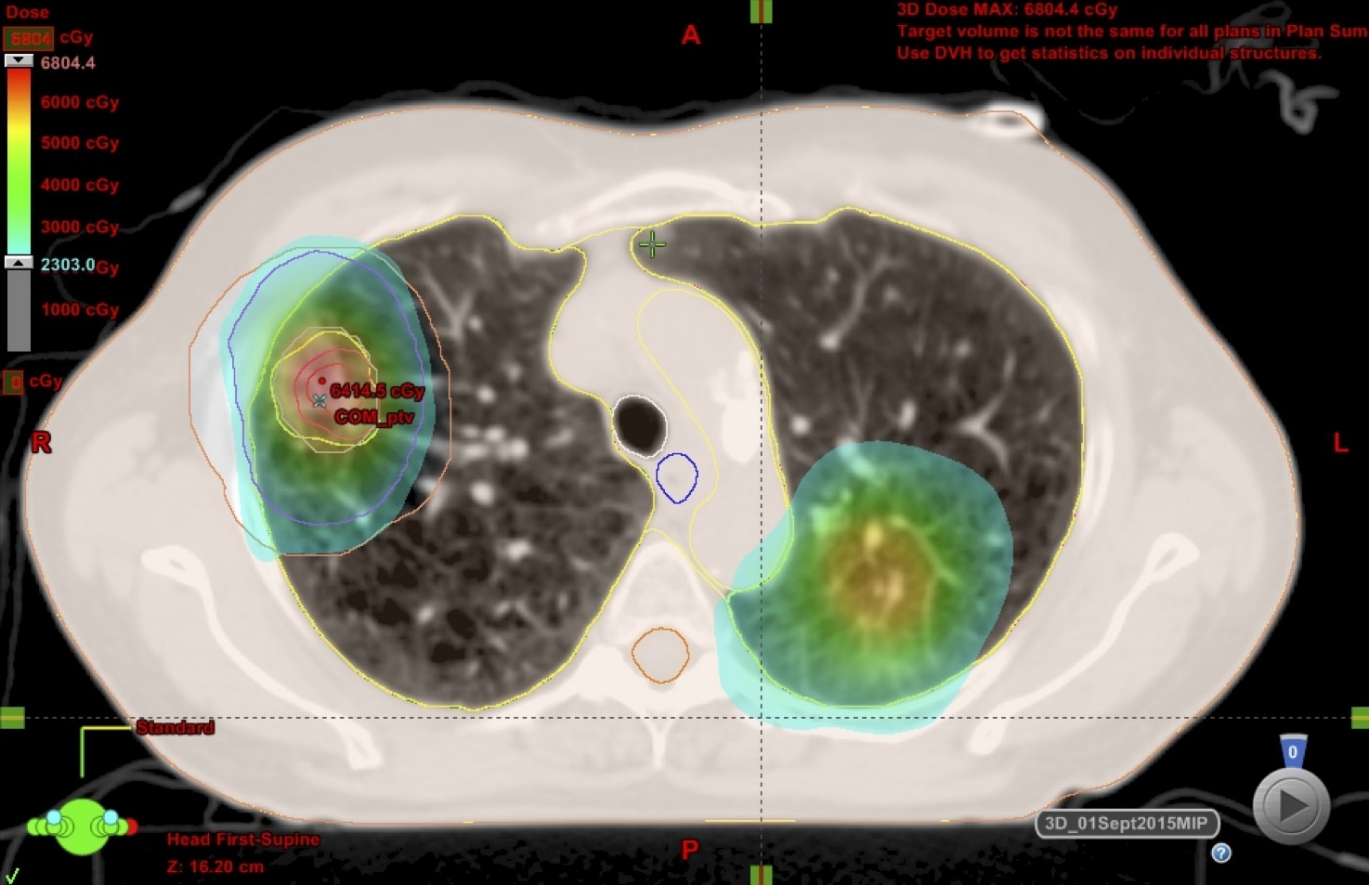
SBRT dose distributions: 48 Gy, prescribed to 95% of the PTV, delivered in four fractions over two weeks, by dynamic conformal arcs.

**Figure 3:**
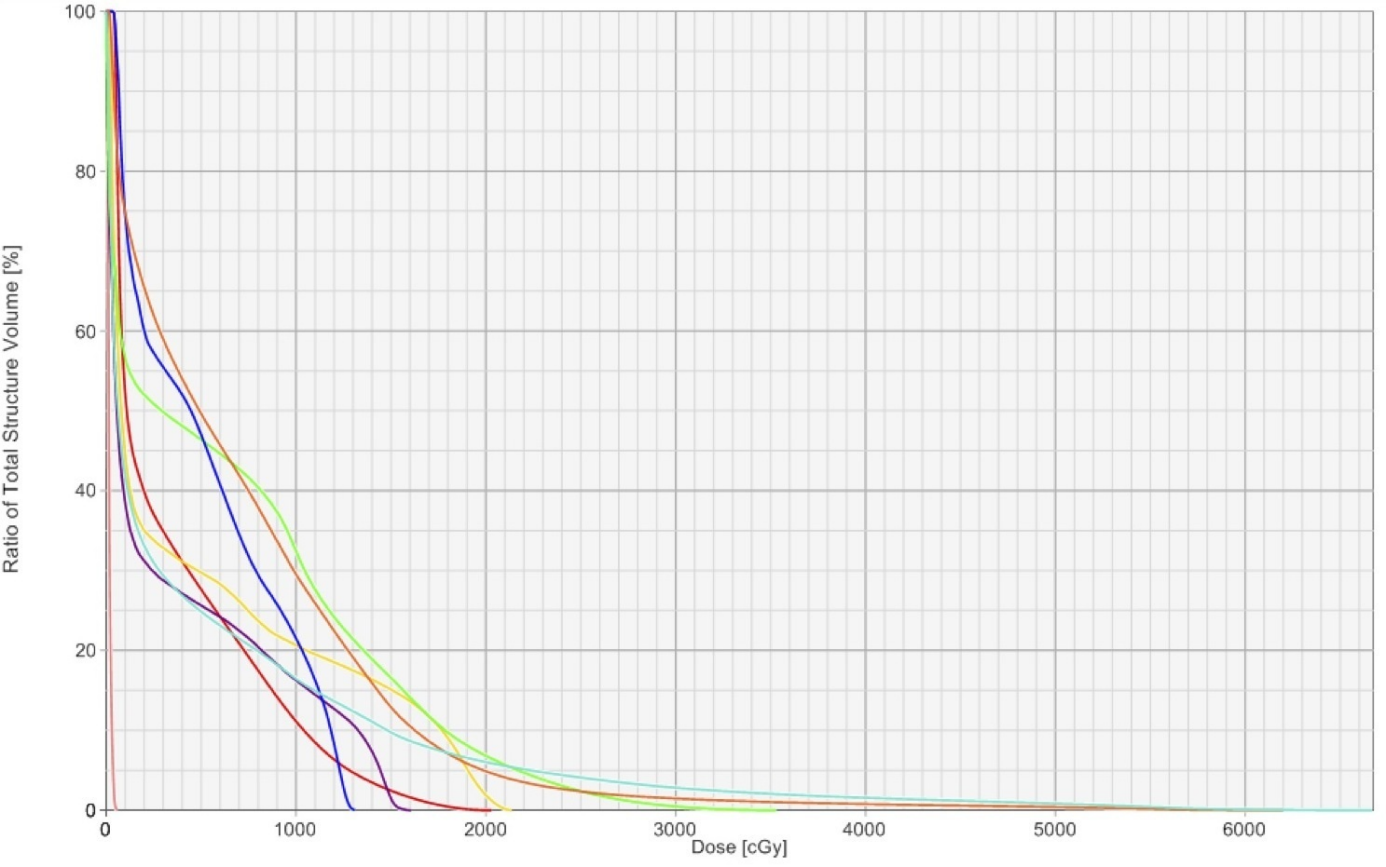
Dose-volume histogram for SBRT plan sum. green = aorta, orange = D 2 cm, dark blue = bronchial tree, yellow = spinal cord, purple = esophagus, light blue = lungs (combined), red = pulmonary artery, pink = heart

The RUL NSCLC was treated with SBRT (48 Gy in four fractions), which the patient tolerated well (Figures 2-3). Post-treatment imaging is pending to assess disease response.

## Discussion

Prior to discussion of treatment, it should be appreciated that staging synchronous or metachronous primary lung cancers requires diagnostic acumen. Several case reports underline the importance of thorough staging, to allow for appropriate treatment to be offered to patients who could be easily mistaken to have multicentric disease or intrapulmonary metastases and thereby be denied radical treatment [8]. For similar reasons, lower survival rates have been associated with a short (< 2 years) interval between detection of first and second primary tumours in metachronous lung cancer [9].

The challenge in this case lay not in the diagnosis but in the limitation of treatment options due to very poor baseline pulmonary function, secondary to severe COPD and advanced emphysema. The patient was assessed to not be a candidate for radical thoracic radiotherapy. As such, the initial proposed treatment was systemic chemotherapy, foregoing the 5.4% increase in three-year survival offered by the combined approach [1]. This was clearly suboptimal, given the low 8.9% three-year survival rate in LS-SCLC treated with chemotherapy alone; however, in light of the patient’s small primary and lack of nodal disease, it was conjectured that she might do better than the population studied [1].

Although SBRT is widely used as a curative treatment for peripheral Stage I NSCLC, few studies have been published on the use of SBRT for SCLC. As SCLC is generally more radiosensitive than NSCLC, SBRT in combination with chemotherapy would seem an effective LS-SCLC treatment, particularly in patients who are not fit to undergo surgical resection or conventionally fractionated radiotherapy. A single-institution report on eight inoperable LS-SCLC patients treated with SBRT (48 Gy in 4 fractions) and chemotherapy demonstrated this to be a safe and effective alternative to surgery and chemotherapy, with three-year survival and local control rates of 72% and 100%, respectively, and minimal toxicity [10].

Further study is necessary to determine the optimal SBRT dose and fractionation schedule for treating LS-SCLC. It has been proposed that, given the relatively higher radiosensitivity of SCLC, a slightly lower radiation dose than that used to treat NSCLC may be appropriate. Furthermore, given the rapid proliferation rate of SCLC, a greater number of SBRT fractions may be of benefit. Finally, ascertaining the optimal timing of chemotherapy with SBRT, be it neoadjuvant, concurrent, or adjuvant, requires further investigation [10].

The primary literature provides better-established recommendations for treating this patient’s NSCLC. For fit patients with Stage I NSCLC, surgery is considered the primary curative treatment. However, severe COPD (Global Initiative for COPD III-IV) presents an increased risk of postoperative complications. For these patients, SBRT is considered a safe alternative first-line treatment, which has been demonstrated to be non-inferior to surgery in one- and three-year survival [11]. Furthermore, when compared to conventionally fractionated radiotherapy in Stage I NSCLC patients, SBRT has been shown to be less expensive and associated with superior local control and overall survival [12]. A poor baseline pulmonary function test, as this patient had, has been observed to not increase pulmonary toxicity or decrease overall survival in Stage I NSCLC after SBRT [13].

In considering what may have predisposed this patient to develop a second primary lung cancer, three risk factors can be identified: COPD, smoking, and SCLC. COPD has been shown to increase the risk of all subtypes of lung cancer, independent of tobacco use, with a suggested mechanism involving genetic changes, cytokines, and cell cycle dysregulation in the presence of proteinases produced by immune cells [14]. Smoking has been hypothesized to increase the risk of synchronous multiple primary lung cancers by inducing a mutation in the p53 tumour suppressor gene; p53 status was not assessed in this case [15].

In patients with SCLC, studies have demonstrated that smoking cessation before or at the time of initial treatment results in a 3- to 4-fold reduction in the relative risk of development of a second primary lung cancer [3]. Evidently smoking cessation should be a component of such patients’ management to decrease their risk of developing a second primary lung cancer, associated with COPD or with smoking itself. Although post-treatment surveillance practices vary and their survival benefit remains questionable, in such a population with ongoing risk factors of COPD and smoking, close surveillance seems warranted, both for monitoring for recurrence of the treated primary and for screening for potentially curable metachronous cancers [9].

## Conclusions

A case of metachronous SCLC and NSCLC has been presented here in a patient with an insufficient pulmonary function to tolerate surgical resection or conventionally fractionated thoracic radiotherapy. The use of consolidation SBRT after chemotherapy to achieve a complete radiologic response was demonstrated in the treatment of LS-SCLC, although it was noted that further research is required to define SBRT dose, fractionation schedule, and timing with chemotherapy in this setting. Careful staging of patients with multiple pulmonary nodules, promotion of smoking cessation to reduce the risk of developing a second primary lung cancer, and appropriate post-treatment surveillance were recognized as important components of management.
